# Positive effects of dietary *Clostridium butyricum* supplementation on growth performance, antioxidant capacity, immunity and viability against hypoxic stress in largemouth bass

**DOI:** 10.3389/fimmu.2023.1190592

**Published:** 2023-08-30

**Authors:** Peijia Li, Xiaoying Chen, Dongqiang Hou, Bing Chen, Kai Peng, Wen Huang, Junming Cao, Hongxia Zhao

**Affiliations:** ^1^ Guangdong Key Laboratory of Animal Breeding and Nutrition, Collaborative Innovation Center of Aquatic Sciences, Institute of Animal Science, Guangdong Academy of Agricultural Sciences, Guangzhou, China; ^2^ College of Fisheries, Guangdong Ocean University, Zhanjiang, China

**Keywords:** *Clostridium butyricum*, Largemouth bass, growth performance, antioxidant status, immune response

## Abstract

The effects of dietary supplementation of *Clostridium butyricum* (CB) on growth performance, serum biochemistry, antioxidant activity, mRNA levels of immune-related genes and resistance to hypoxia stress were studied in largemouth bass. Feed with CB0 (control, 0 CFU/kg), CB1 (4.3×10^8^ CFU/kg), CB2 (7.5×10^8^ CFU/kg), CB3 (1.5×10^9^ CFU/kg) and CB4 (3.2×10^9^ CFU/kg) CB for 56 days, and then a 3 h hypoxic stress experiment was performed. The results showed that dietary CB significantly increased the WGR (weight gain rate), SGR (specific growth rate), PDR (protein deposition rate) and ISI (Intestosomatic index) of largemouth bass (*P<*0.05). Hepatic *GH* (growth hormone)/*IGF-1* (insulin-like growth factor-1) gene expression was significantly upregulated in the CB3 and CB4 groups compared with the CB0 group (*P<*0.05), while the FC (feed conversion) was significantly decreased (*P<*0.05). Serum TP (total protein) and GLU (glucose) levels were significantly higher in the CB4 group than in the CB0 group (*P<*0.05), while the contents of serum AST (aspartate transaminase), ALT (alanine transaminase), AKP (alkline phosphatase) and UN (urea nitrogen) in CB4 were significantly lower than those in CB0 (*P<*0.05). T-AOC (total antioxidant capacity), SOD (superoxide dismutase), CAT (catalase), POD (peroxidase) and GSH-Px (glutathione peroxidase) activities were significantly higher in CB3 and CB4 groups than in CB0 group (*P<*0. 05). The liver MDA (malondialdehyde) content of CB1, CB2, CB3 and CB4 groups was significantly higher than that of CB0 group (*P<*0. 05). The relative expressions of *IL-1β* (interleukin 1β), *TNF-α* (tumor necrosis factor α) and *TLR22* (toll-like receptor-22) genes in CB2, CB3 and CB4 groups were significantly lower than those in CB0 group (*P<*0.05). The relative expression of *IL-8* (malondialdehyde) and *MyD88* (Myeloid differentiation factor 88) genes in the CB4 group was significantly lower than that in the CB0 group (*P<*0.05). The liver LZM (lysozyme) content of CB2, CB3 and CB4 groups was significantly higher than that of CB0 group (*P<*0. 05). The relative expression of *IL-10* (interleukin 10) and *TGF-β* (transforming growth factor β) genes in the CB4 group was significantly higher than that in the CB0 group (*P<*0.05). Under hypoxic stress for 3 h, the CMR of CB0 group was significantly higher than that of CB1, CB2, CB3 and CB4 groups (*P<*0.05). Dietary CB can improve the growth performance and resistance to hypoxic stress of largemouth bass by regulating the expression of *GH*/*IGF-1* gene and inflammatory factors and inhibiting *TLR22*/*MyD88* signaling pathway.

## Graphical abstract

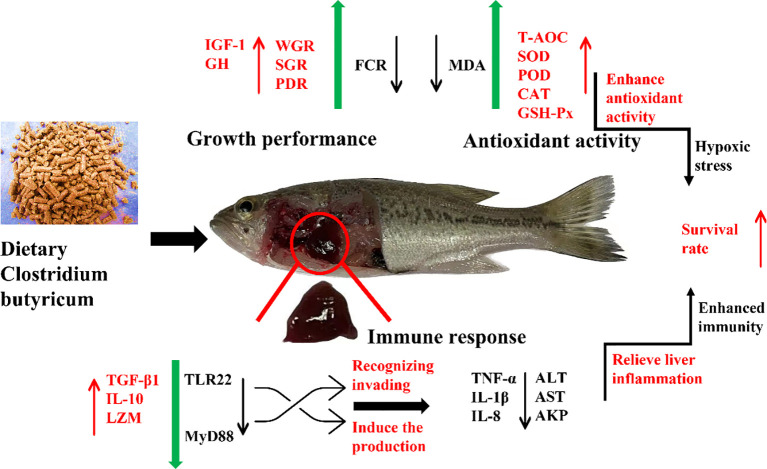

## 1 Introduction

The extensive use of antibiotics in intensive farming to control bacterial diseases exacerbates the development and spread of antibiotic resistance (1). The incidence of infectious diseases caused by antibiotic-resistant bacteria is currently increasing, resulting in a higher mortality rate than cancer (2). The misuse of antibiotics results in microbial dysbiosis and the emergence of antimicrobial resistance, thereby posing significant public health challenges (3, 4). Consequently, the utilization of antibiotics as feed additives was prohibited in the European Union, United States, and China in 2006, 2014, and 2020 correspondingly (5). Therefore, it is imperative to explore alternatives that can substitute antibiotics. Probiotics are defined as live microorganisms that optimize the microbial balance and promote health (6). Probiotics have been successfully used in farmed freshwater or marine fish, including Nile tilapia (*Oreochromis niloticus*), Javanese carp (*Puntius gonionotus*), and rainbow trout (*Oncorhynchus mykiss*), among others (7–9). Probiotics are the preferred therapeutic agents for managing inflammatory disorders, such as diarrheal disease and inflammatory bowel disease. A plethora of studies have demonstrated that dietary supplementation with probiotics can significantly augment fish health (5, 10–12).

*Clostridium butyricum* (CB) is a Gram-positive bacterium that exerts beneficial effects on growth promotion, inflammation suppression, and pathogenic bacteria reduction (13, 14). In recent years, CB has gained widespread application in the livestock and aquatic animal industries, primarily for enhancing growth performance and bolstering disease resistance (15, 16). Additionally, due to its ability to withstand low pH and high temperatures, CB is frequently utilized in fish as a preventative measure against fish pathogens or antibiotic resistance (17). Studies have shown that the addition of CB in the range of 10^7^ - 10^11^ CFU/kg significantly improved the growth performance of silver pomfret (*Pampus argenteus*) (18), large yellow croaker (*Larimichthys crocea*) (19), and Nile tilapia (20), enhanced the immune response of giant freshwater prawn (Macrobrachium rosenbergii) (21), and increased the antioxidant activity of kuruma shrimp (*Marsupenaeus japonicas*) (22). However, the impact of dietary supplementation with CB on growth, antioxidant activity, immune response and hypoxic stress resistance in largemouth bass remains uncertain.

Largemouth bass (*Micropterus salmoides*), a freshwater farmed fish, commands high market value owing to its rapid growth and delectable flesh (23). Meanwhile, due to its immense popularity among Chinese consumers, it constitutes a significant portion of China’s breeding industry (24, 25). However, nutritional deficiencies, high densities, and environmental changes often lead to metabolic disorders in fish, which can result in reduced disease resistance and increased susceptibility to disease outbreaks (26). Dietary nutrients and supplementation significantly boost growth of Largemouth bass (27–29). Studies have demonstrated that probiotics have been efficaciously employed in numerous aquaculture species to stimulate growth, optimize feed utilization, and augment organismal resistance against diseases (30). Revised sentence: However, there is a lack of research on the utilization of CB as a feed additive for largemouth bass, and the mechanisms underlying CB’s promotion of growth and enhancement of disease resistance remain unclear. The objective of this investigation was to examine the impact of CB on growth performance, antioxidant capacity, immune response, and hypoxic stress in juvenile largemouth bass.

## 2 Materials and methods

### 2.1 Experimental diets

The CB with a count of 8×10^8^ colony-forming units (colony-forming units, CFU)/ml was obtained from China Organic Biotechnology Co., Ltd., China. Juvenile largemouth bass were fed with five isonitrogenous (50%) and isolipidic (9%) diets, which were supplemented with CB at 0 (CB0), 2.5 (CB1), 5 (CB2), 10 (CB3), and 20 (CB4) ml/kg of diet, respectively. The final CB concentrations in the five diets were 0, 4.3×10^8^, 7.5×10^8^, 1.5×10^9^ and 3.2×10^9^ CFU/kg, which were determined by the plate count method (31). The raw materials were weighed in accordance with the feed formulation, crushed, and sieved. Prior to mixing into the raw materials, the CB solution was mixed with water. All diets were prepared and pelleted into 1.5 mm diameter by twin screw extruder (SLX-80, South China University of Technology, China). Dry at 55°C for 6 h and store the feed at -20°C after packaging in groups. The ingredients and proximate composition of experimental diets are presented in **Table 1**.

**Table 1:** Composition and approximate composition of the experimental rations (in dry matter, %).

Item	Group				
	CB0	CB1	CB2	CB3	CB4
Ingredients					
Fish meal (Peru, crude protein 67.7%) ^a^	35.00	35.00	35.00	35.00	35.00
Soy protein concentrate (crude protein 64.6%) ^a^	9.00	9.00	9.00	9.00	9.00
Blood meal ^a^	3.00	3.00	3.00	3.00	3.00
Shrimp shell meal ^a^	5.00	5.00	5.00	5.00	5.00
Soybean meal^a^	20.00	20.00	20.00	20.00	20.00
Cottonseed protein meal^a^	9	9	9	9	9
Tapioca meal^a^	9	9	9	9	9
Fish oil^a^	3.00	3.00	3.00	3.00	3.00
Soybean oil^a^	3.00	3.00	3.00	3.00	3.00
Vitamin premix^b^	1.00	1.00	1.00	1.00	1.00
Mineral premix^c^	1.00	1.00	1.00	1.00	1.00
Ca(H_2_PO4)_2_ ^a^	1.00	1.00	1.00	1.00	1.00
Choline chloride^a^	0.20	0.20	0.20	0.20	0.20
Sodium alginate^a^	0.80	0.80	0.80	0.80	0.80
*Clostridium butyricum*	0.00	0.25	0.50	1.00	2.00
Total	100.00	100.00	100.00	100.00	100.00
Nutrients compositions of experimental diets (in dry matter, %)					
*Clostridium butyricum*	0	4.3×10^5^	7.5×10^5^	1.5×10^6^	3.2×10^6^
Crude protein	50.55	50.63	50.96	50.21	50.35
Crude lipid	9.63	9.40	9.45	9.03	9.49
Ash	12.44	12.45	12.53	12.47	12.52
Moisture	3.73	4.20	3.46	4.14	3.64

a Fishtech Fisheries Science & Technology Company, LTD, Institute of Animal Science, Guangdong Academy of Agricultural Sciences (Guangzhou, China).

b Vitamin premixes: VA 4,000,000 IU, VD_3_ 2,000,000 IU, VE 30 g, VK_3_ 10_ g_, VB_1_ 5 g, VB_2_ 15 g, VB_6_ 8 g, VB_12_ 0.02_ g_, nicotinic acid 40 g, calcium pantothenate 25 g, folic acid 2.5 g, inositol 150 g, biotin 0.08 g. Moisture ≤ 10%.

c Mineral premixes: MgSO_4_·H_2_O 12 g, KCl 90 g, Met-Cu 3 g, FeSO_4_·H_2_O 1 g, Ca (IO_3_)_2_ 0.06 g, Met-Co 0.16 g, ZnSO_4_·H_2_O 10 g, NaSeO_3_ 0.003 6 g. Moisture ≤ 10%.

### 2.2 Feeding and management

This feeding experiment was carried out in an indoor recirculating aquaculture system in the aquatic laboratory of the Institute of Animal Science, Guangdong Academy of Agricultural Sciences (Guangzhou, China) and lasted for 56 d. The fish were purchased from Guangzhou, China, and were acclimated with the control diet for 1 week in an indoor recirculating aquaculture system. Six hundred fish (5.02 ± 0.01 g) were stocked into fifteen cylindrical fiberglass tanks (water volume 300 L) at 40 fish per tank to conduct the experiment. Five experimental diets were randomly allocated to triplicate groups of fish. During the 56-day feeding trial, the water temperature ranged from 25 to 32°C, pH 7.8-8.0, and dissolved oxygen > 8.0 mg/L. Fish were reared under 12 h light: 12 h dark dialcycle photoperiod. All the fish were manually fed to satiation with the experimental diets two times daily (8:30 and 18:30) (initially 5% to 6% of body weight per day and then gradually increased).

### 2.3 Sample collection and analysis

After the end of the 8-week feeding experiment, the fish were fasted for 24 h, and the final body weight of each cage was weighed, and the survival rate (SR) was calculated. Eighteen fish were randomly selected from each replicate and anesthetized in 120 mg/L tricaine methanesulfonate (MS-222) solution, and 3 fish were stored at -20 °C for the determination of routine nutrient composition of whole fish. The body weight, body length, intestines weight and viscera weight of six fish were measured to calculate weight gain rate (WGR), specific growth rate (SGR), protein deposition rate (PDR), feed conversion (FC), intestosomatic index (ISI) and condition factor (CF). Blood samples were collected from the tail vein of 9 fish, left at room temperature for 4 h, centrifuged at 3500 r/min for 10 min, and the supernatant was taken to prepare serum and stored at -80° C until use. The blood of 9 fish was collected and placed on ice for rapid dissection, and the liver of 3 fish was determined for immune antioxidant indexes. Livers from three fish were fixed in 10% formalin solution and stored at room temperature to produce liver paraffin sections. The routine nutrient composition of experimental diets and whole fish was determined as follows: Water content was determined by oven drying to constant weight at 105 °C (GB/T 6435-2014), crude protein content (N×6.25) was determined by semi-automatic Kjeldner nitrogen determination method (GB/T 6432-2018), crude fat content was determined by ether extraction method (GB/T 6433-2006), crude ash content was determined by the method of burning to constant weight at 550 °C (GB/T 6433-2006).

### 2.4 Serum biochemical analysis

After the fish were anesthetized, blood was collected from the tail vein, and the blood samples were centrifuged (3500 × g, 10 min, 4°C) to separate the serum. Serum total protein (TP) content was determined by biuret method. alanine transaminase (ALT) activity was measured by spectrophotometry. glucose (GLU) content was measured by glucose oxidase method. UN), triglyceride (TG) and aspartate transaminase (AST) activity were measured by enzyme coupling rate method, enzymatic method and spectrophotometric method, respectively. High-density lipoprotein cholesterol (HDL), low-density lipoprotein cholesterol (LDL), The contents of total cholesterol (TCHO) and alkaline phosphatase (AKP) were determined by enzymatic method. 1. The Hitachi 7600 fully automated analyzer was utilized to measure all parameters at Guangzhou Jinyu Medical Testing Center (Guangzhou, China).

### 2.5 Enzyme activity analysis

The liver samples were added with normal saline according to the ratio of weight liver: volume of normal saline (1:9), and the liver samples were fully broken under the condition of ice water bath. After the broken liver samples were centrifuged for 10 min (2500 r/min, 4 °C), the supernatant was taken after centrifugation for enzyme activity detection. The activity of peroxidase (POD) was determined by A084-2 colorimetric method, the activity of Lysozyme (LZM) was determined by A050 turbidimetric method, and the activity of superoxide dismutase was determined by superoxide dismutase. SOD activity was determined by A001-1 hydroxylamine method, catalase (CAT) activity was determined by A007-1 visible light method, total antioxidant capacity, T-AOC capacity was determined by A015 colorimetric method. Content of malondialdehyde (MDA) and activity of glutathione peroxidase (GSH-Px) were determined by A003-1 TBA method. The enzyme activity kit was purchased from Nanjing Jiancheng Bioengineering Institute (Nanjing, China), and the measurement procedure, principle and calculation formula were referred to the kit manual.

### 2.6 qRT-PCR analysis

Total RNA was extracted from the whole liver of largemouth bass using RNA Isolation Kit (Vazyme, Nanjing, China), following the protocol of the manufacturer, and electrophoresed on a 1.2% denaturing agarose gel to test the integrity. The RNA reverse was then transcribed to cDNA using HiScript®III RT SuperMix for qPCR kit (Vazyme, Nanjing, China). The qPCR assay was carried out using ChamQ Universal SYBQ qPCR Master Mix kit (Vazyme, Nanjing, China). The amplification was carried out in a 20 μL reaction volume containing 10 μL SYBQ Green Master Mix, 0.4 μL of each respective primer (10 μmol/L), 2 μL cDNA product, and 7.2 μL RNA-free water. The specific primers and primer sequences of the housekeeping gene (*β-actin*) are shown in **Table 2**, and all primers were synthesized by Shanghai Sangon Biotechnology Co., LTD. (Shanghai, China). *β-actin* was used as a nonregulated reference gene to normalize target gene transcript levels in largemouth bass studies, furthermore, *β-actin* gene expression of the intestine was also stable and was not significantly affected by dietary CB in our present research. All reactions were performed in duplicate, and each assay was repeated three times. The gene expression levels were analyzed using the 2^−ΔΔCT^ method (32).

**Table 2:** Primer sequences.

Genes	Sequence Information		
	Forward primer (5′-3′)	Reverse primer (5′-3′)	GenBank
*β-actin*	GGACACGGAAAGGATTGACAG	CGGAGTCTCGTTCGTTATCGG	XM_038695351.1
*IL-1β*	CGTGACTGACAGCAAAAAGAGG	GATGCCCAGAGCCACAGTTC	XM_038696252.1
*IL-8*	CGTTGAACAGACTGGGAGAGATG	AGTGGGATGGCTTCATTATCTTGT	XM_038704088.1
*IL-10*	CGGCACAGAAATCCCAGAGC	CAGCAGGCTCACAAAATAAACATCT	XM_038696252.1
*TNF-α*	CTTCGTCTACAGCCAGGCATCG	TTTGGCACACCGACCTCACC	XM_038710731.1
*TGF-β*	GCTCAAAGAGAGCGAGGATG	TCCTCTACCATTCGCAATCC	XM_038693206.1
*TLR22*	TCGCTGTTCACCAATCTG	TAGTTCTCCTCTCCATCTGT	MN807054.1
*MyD88*	CTCAACCCCAAGAACACA	CGAAGATCCTCCACAATG	XM_038728827.1
*IGF-I*	CTTCAAGAGTGCGATGTGC	GCCATAGCCTGTTGGTTTACTG	DQ666526
*GH*	CCCCCAAACTGTCAGAACT	ACATTTCGCTACCGTCAGG	DQ666528

The cDNA sequence of the target gene from NCBI (National Center for Biotechnology Information) was used, and the primer sequence was designed using primer 6, and then synthesized at Shanghai Biotechnology Co.

IL1β, interleukin 1β; IL8, interleukin 8; IL10, interleukin 10; TNF-α, tumor necrosis factor α; TGF-β, transforming growth factor β1; TLR22, toll-like receptor 2; MyD88, myeloid differentiation primary response gene 88; IGF-I, insulin-like growth factor-I; GH, growth hormone.

### 2.7 Hypoxia stress test

At the end of the culture experiment, 16 fish were collected from each experimental group and subjected to hypoxic stress experiments according to the method described by Zeng et al. (33). Drain the water in the tank to the water surface to the bottom 10 cm of the tank (approximately 50 L), cover the tank with transparent plastic film and stop oxygen supply, causing an oxygen deficient environment. Dissolved oxygen (DO) was measured at 30-minute intervals during the experiment (Seven2Go, Mettler Toledo, USA). The DO level decreased from 7.6 mg L^-1^ to 0 mg L^-1^ after 1.5 h of continuous testing and remained at this level. When the cumulative mortality rate (CMR) of the control group (CB0) reached 50%, the experiment was stopped. Observation of the experimental fish was confirmed as a dead state when it did not respond to external stimuli. Fasting was done during the trial, and the number of fish stocks was counted to calculate the CMR after the trial.

### 2.8 Statistical analysis

SPSS 25.0 software (Chicago, USA) was used for statistical analysis. One-way analysis of variance (ANOVA) and multipole difference test (Tukey’s test) were used to determine significant differences (*P*< 0.05). Data are expressed as mean ± standard error (SEM).

### 2.9 Calculations

The parameters are calculated according to the following formula:


$$\text{Survival rate}\left(\text{SR},\;\%\right)=100\times {\text{F}}_{\text{final}}/{\text{F}}_{\text{initial}}.$$

$$\text{Weight gain rate}\left(\text{WGR},\;\%\right)=100\times ({\text{W}}_{\text{final}}-{\text{W}}_{\text{initial}})/{\text{W}}_{\text{initial}}.$$

$$\text{Feed conversion}\left(\text{FC}\right)={\text{D}}_{\text{total}}/({\text{W}}_{\text{final}}+{\text{W}}_{\text{dead}}-{\text{W}}_{\text{initial}}).$$

$$\text{Protein deposition rate}\left(\text{PDR},\;\%\right)=100\;\times ({\text{W}}_{\text{final}}\times {\text{CP}}_{\text{final}}-{\text{W}}_{\text{initial}}\times {\text{CP}}_{\text{initial}})/(\text{D}\times {\text{CP}}_{\text{feed}}).$$

$$\text{Specific growth rate}\left(\text{SGR},\;\%\right)=100\times ({\text{LnW}}_{\text{final}}-{\text{LnW}}_{\text{initial}})/\text{T}.$$

$$\text{Condition factor}\left(\text{CF},\text{ g}/{\text{cm}}^{3}\right)=100\times \text{W}/{\text{L}}^{3}.$$

$$\text{Intestosomatic index}\left(\text{ISI},\;\%\right)=100\times {\text{W}}_{\text{intestinal}}/\text{W}.$$

In the formula: F _initial_ is the initial mantissa; F _final_ is the final mantissa; W is the body weight; L is the body length; W _initial_ is the initial fish weight; W _final_ is the final fish body weight; W _dead_ is the dead fish weight; CP _initial_ is the protein content of the initial fish body; CP _final_ is the protein content of the final fish; CP _feed_ is the protein content of the feed; D _total_ is the total weight of the feed; D is the feed intake; T is the breeding time; W _intestinal_ is the intestinal weight.

## 3 Results

### 3.1 Growth performance

**Figure 1:**
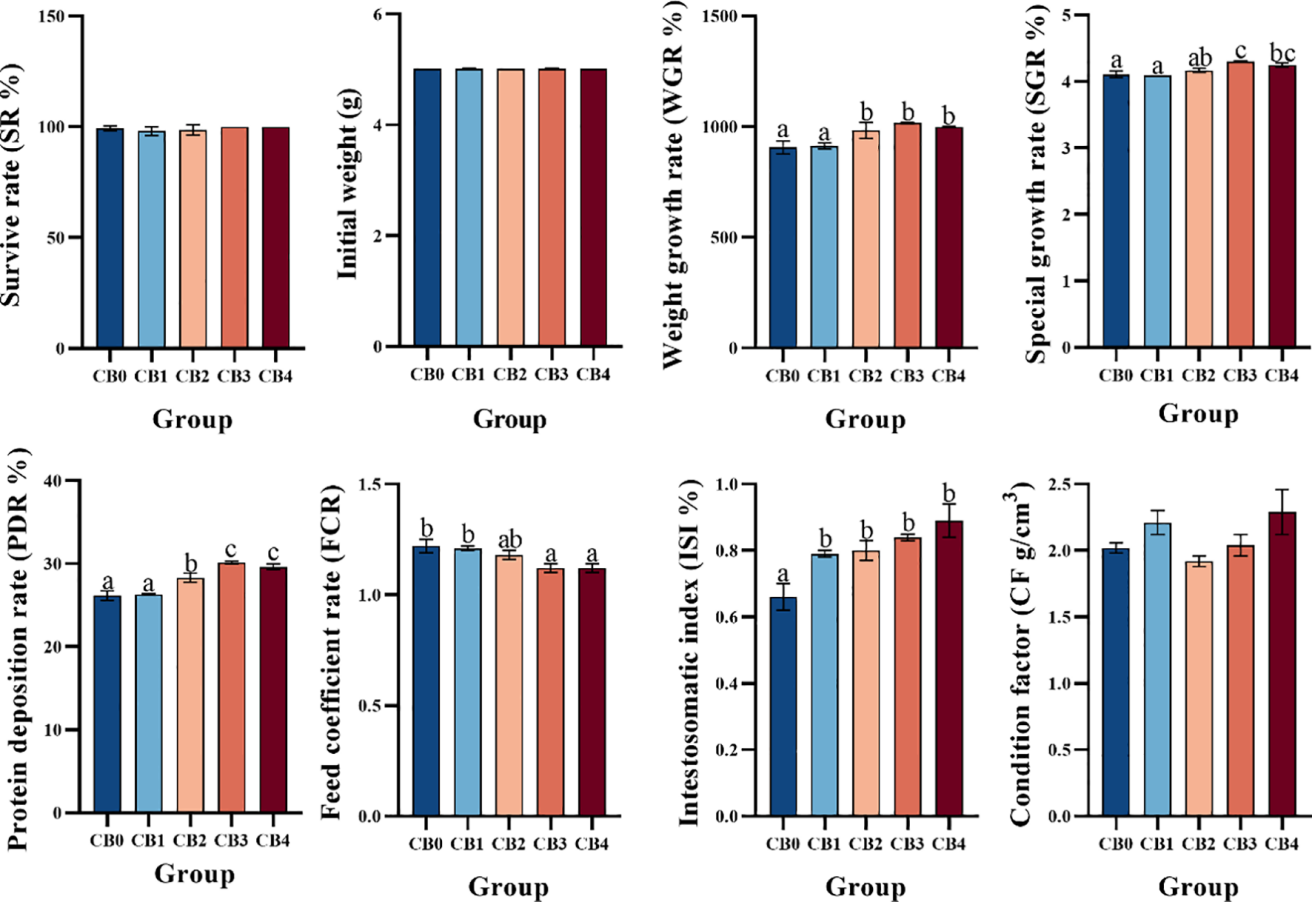
Effects of dietary CB growth performance, feed efficiency, and intestinal growth of juvenile largemouth bass *Micropterus salmoides*. ^a,b,c^ Bars with different superscripts represent significant difference (*P<* 0.05). Data presented are means ± SEM of 3 replicates. The same picture below.

The growth performance is shown in **Figure 1**. WGR and PDR were significantly higher in CB2, CB3 and CB4 groups than in CB0 group (*P*< 0.05). The SGR of CB3 and CB4 groups was significantly higher than that of CB0 group (*P*< 0.05). ISI was significantly higher in CB1, CB2, CB3, and CB4 groups than in CB0 group (*P*< 0.05). FC was significantly lower in CB3 and CB4 groups than in CB0 group (*P*< 0.05). SR was 100% in all groups, and the difference between groups was not statistically significant (*P >*0.05). No significant change in CF between groups (*P >*0.05).

### 3.2 Liver GH-IGF system

**Figure 2:**
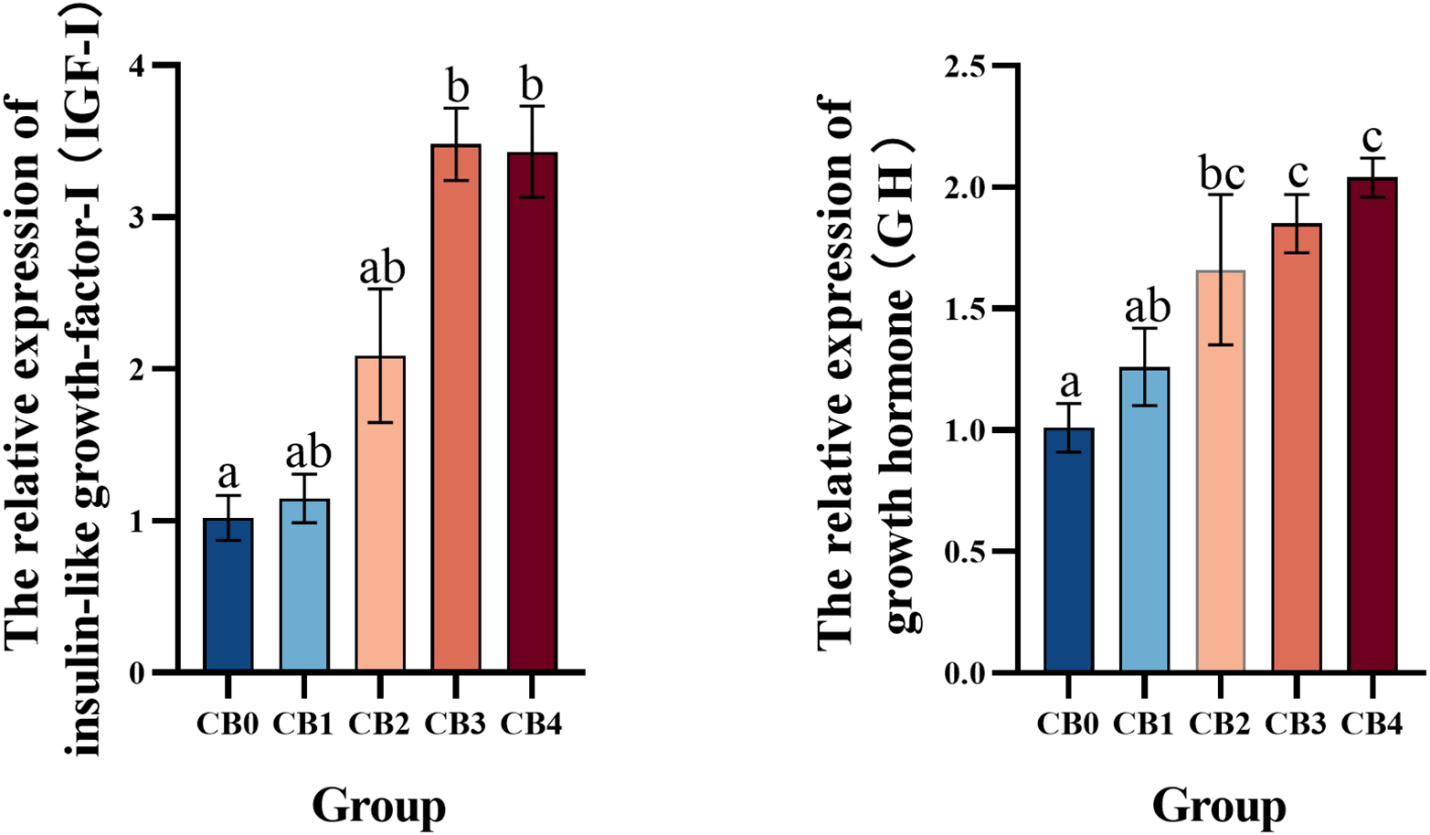
Effect of dietary CB levels on the relative expression of GH and IGF-I in the liver of largemouth bass.

GH-IGF signaling pathway mRNA expression is shown in **Figure 2**. The IGF-I mRNA expression levels in CB3 and CB4 groups were significantly higher than those in CB0 group (*P*< 0.05). The expression of GH mRNA in CB2, CB3, and CB4 was significantly higher than that in CB0 (*P*< 0.05).

### 3.3 Serum biochemistry

**Figure 3:**
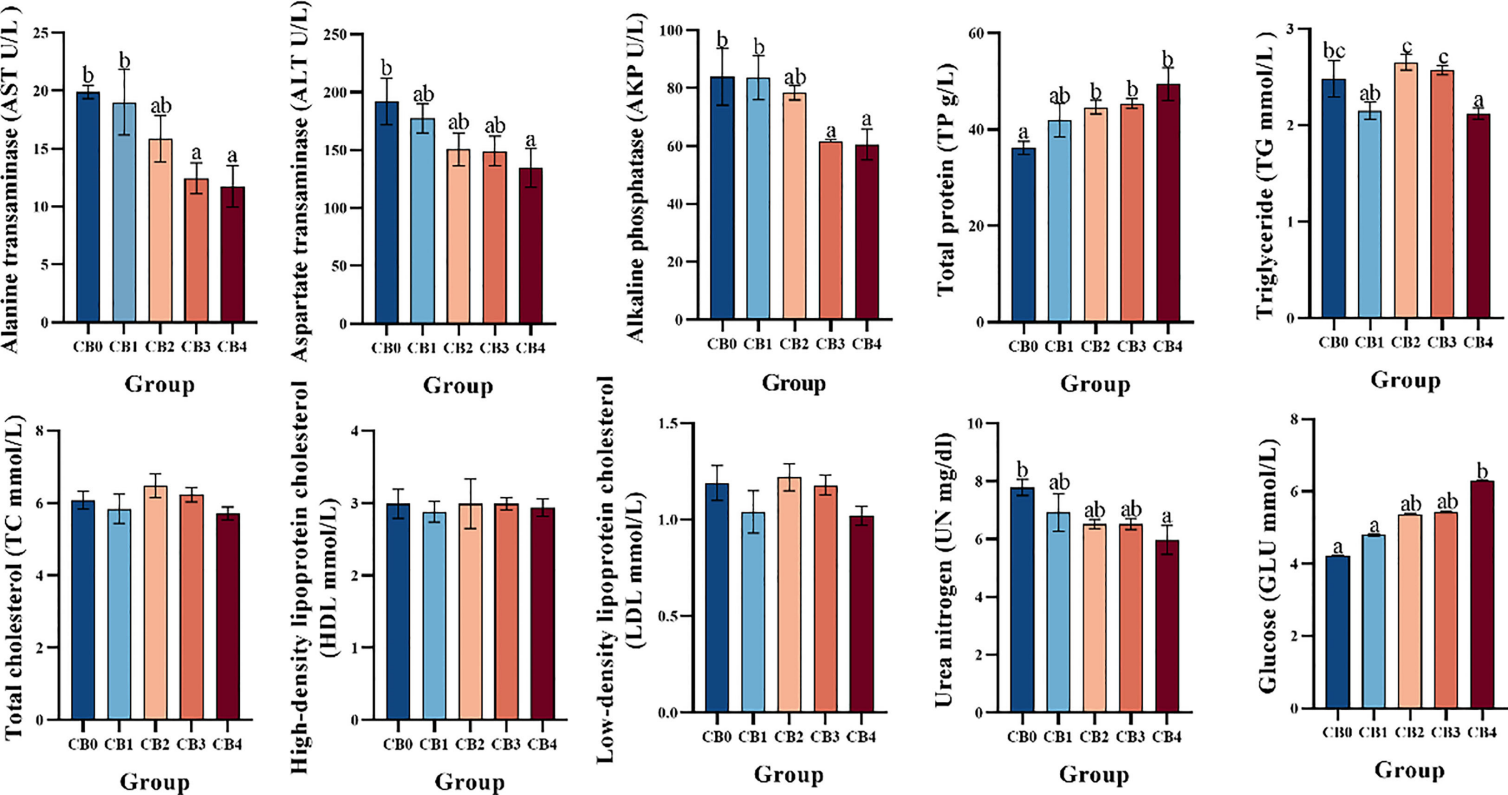
Effect of dietary CB on serum biochemistry of largemouth bass.

The serum biochemical indices are shown in **Figure 3**. AST and AKP were significantly lower in CB3 and CB4 groups than in CB0 group (*P*< 0.05). ALT, UN and TG were significantly lower in the CB4 group than in the CB0 group (*P*< 0.05). CB2, CB3 and CB4 serum TP levels were significantly higher than CB0 (*P*< 0.05). No significant changes between groups in TC, HDL, LDL (*P >*0.05).

### 3.4 Antioxidative parameters

**Figure 4:**
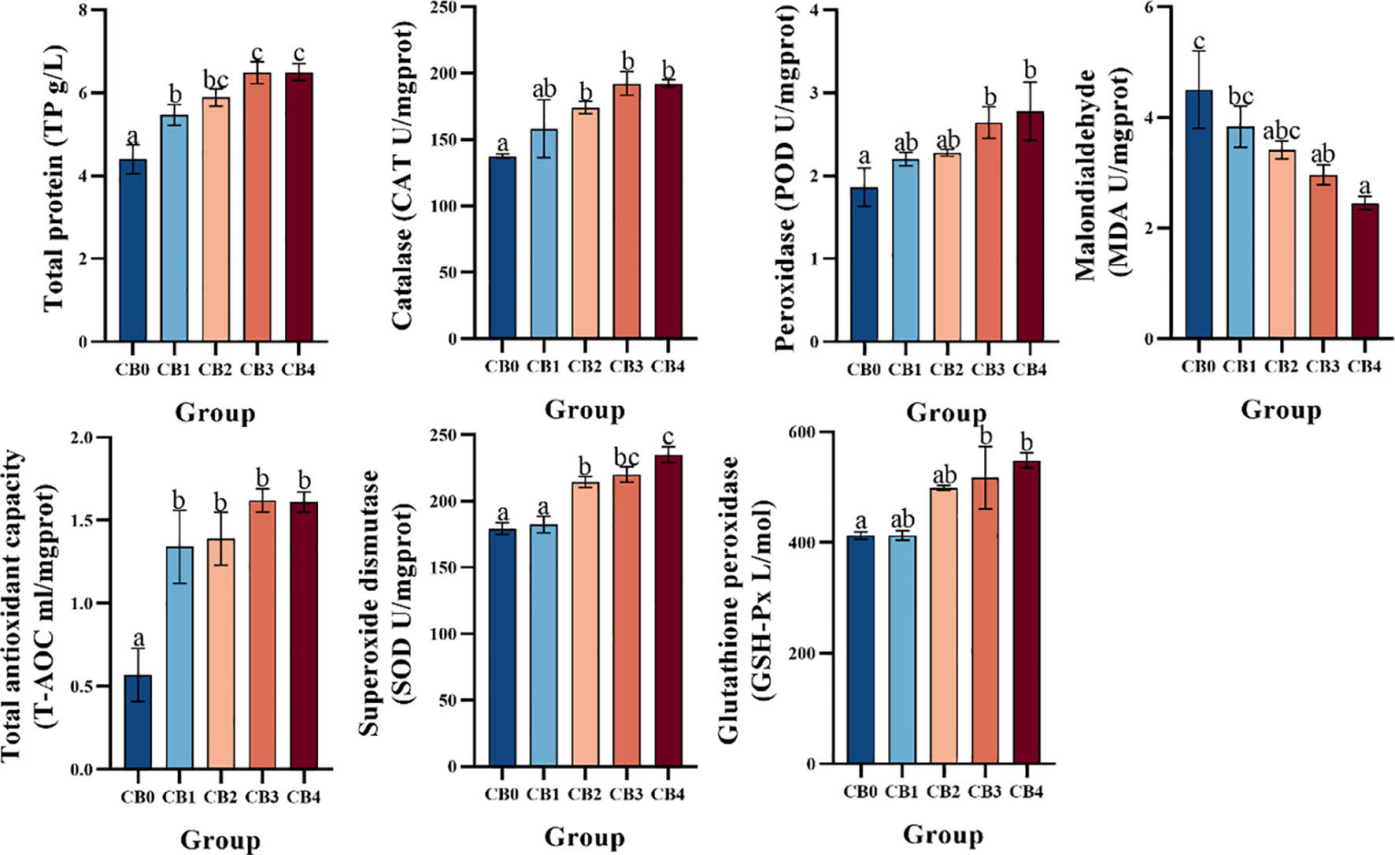
Effect of dietary CB on antioxidant activity in the liver of largemouth bass.

Antioxidant activity is shown in **Figure 4**. TP and T-AOC were significantly higher in CB1, CB2, CB3 and CB4 groups than in CB0 group (*P*< 0.05). CAT and SOD were significantly higher in CB2, CB3 and CB4 groups than in CB0 group (*P*< 0.05). POD and GSH-Px were significantly higher in CB3 and CB4 than in CB0 (*P*< 0.05). The MDA of CB3 and CB4 groups was significantly lower than that of CB0 group (*P*< 0.05).

### 3.5 Immune parameters 

**Figure 5:**
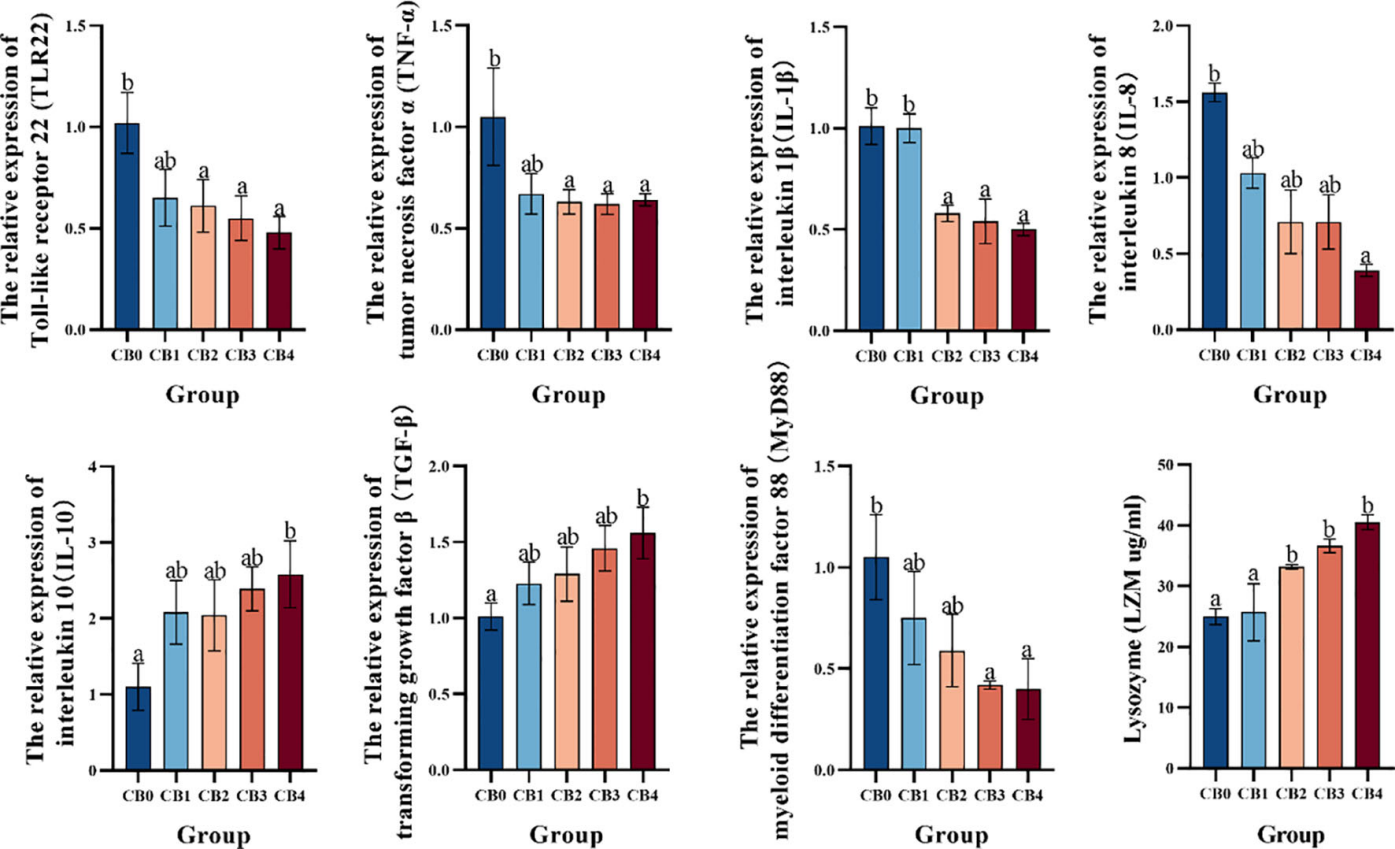
Effect of dietary CB on hepatic immune genes in the liver of largemouth bass.

Immune-related gene expression is shown in **Figure 5**. *TLR22*, *TNF-α* and *IL-1β* were significantly lower in CB2, CB3 and CB4 groups than in CB0 group (*P*< 0.05). *IL-8* was significantly lower in CB4 than in CB0 (*P*< 0.05). *MyD88* was significantly lower in CB3 and CB4 groups than in CB0 group (*P*< 0.05). *IL-10* and *TGF-β* were significantly higher in the CB4 group than in the CB0 group (*P*< 0.05). LZM was significantly higher in CB2, CB3 and CB4 groups than in CB0 group (*P*< 0.05).

### 3.6 Hypoxic stress test

**Figure 6:**
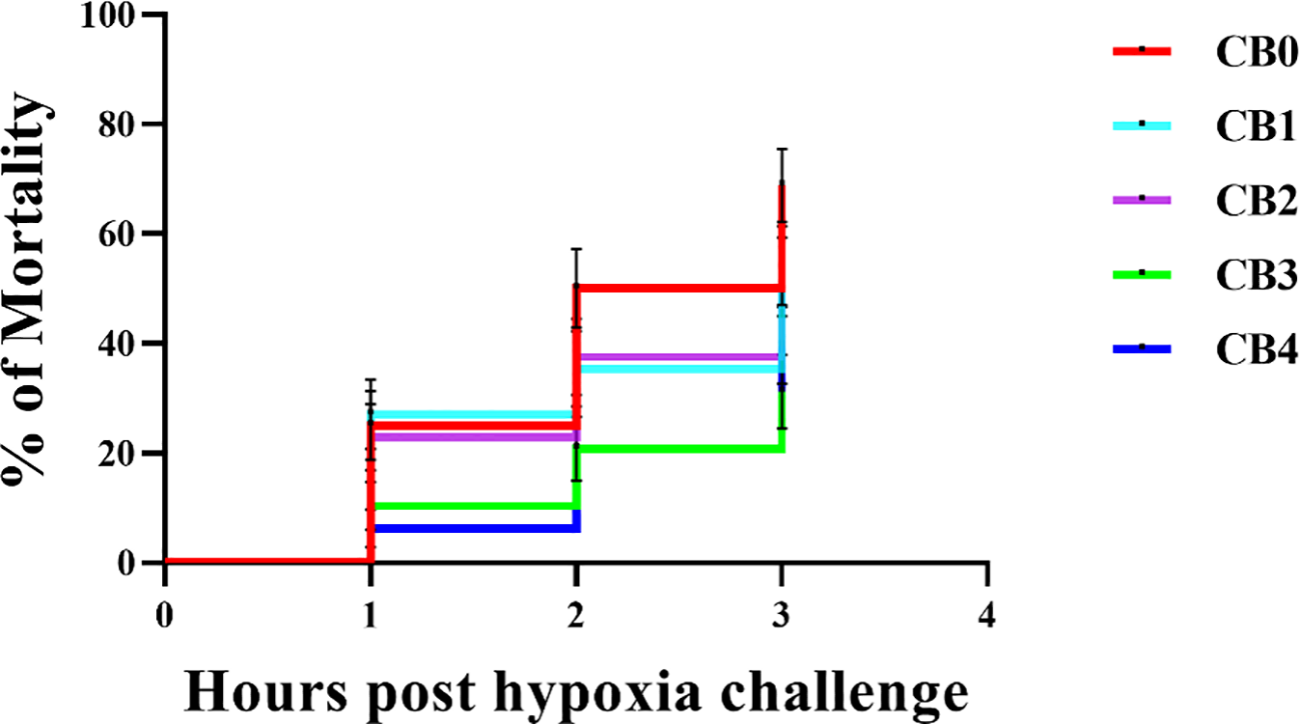
Mortality of largemouth bass *Micropterus salmoides* after 3 hours of hypoxic stress.

Hypoxic stress mortality is shown in **Figure 6**. CMR of largemouth bass significantly increased 3 hours into the experiment. The CMRs of fish were 68.75% (CB0 DO 0), 54.17% (CB1 DO 0), 52.08% (CB2 DO 0), 31.25% (CB3 DO 0) and 39.58% (CB4 DO 0) after 3 h.

## 4 Discussion

Dietary supplementation with CB significantly enhanced the growth performance of largemouth bass, as evidenced by increased WGR, SGR, ISI, and PDR and decreased FC. Consistent with our findings, CB has been observed to have beneficial effects on the growth of various livestock and fish species, including broilers (16), weaned piglets (34), Pacific white shrimp (*Penaeus vannamei*) (35), Yellow Catfish(*Pelteobagrus fulvidraco*) (36), large yellow croaker (19), tilapia (*Oreochromis niloticus*) (4), and silver pomfret (*silver pomfret*) (18). The *GH*-*IGF-I* signaling pathway is an endocrine signaling pathway that regulates growth and responds to the body’s nutrient metabolism (37–39). The liver is also highly responsive to the nutritional status of the host and exerts significant regulatory effects on host nutrient metabolism and immune function (40–42). The findings of this investigation indicate that the inclusion of CB in the diet significantly upregulated *GH* and *IGF-I* mRNA expression, with *GH* and *IGF-I* being particularly responsive to varying levels of CB. This promotion of *GH* and *IGF-I* mRNA expression by dietary CB may account for its growth-promoting effects on largemouth bass.

For largemouth bass, it is equally important to ensure their health and optimize growth performance. Blood biochemical indicators can provide valuable insights into the fish’s overall health status, nutritional condition, and adaptability to its environment (43–46). Therefore, the effect of dietary additives on blood biochemical indicators must be verified before application. The content of TP in blood plays a crucial role in maintaining normal osmotic pressure and pH of blood vessels, serving as the basis for protein metabolism intake by the body (47). The findings of this study demonstrate that dietary CB can significantly elevate the serum total protein (TP) and glucose (GLU) levels in largemouth bass, which is consistent with the growth trend of protein deposition efficiency in fish. This suggests that CB supplementation can effectively enhance nutrient metabolism and promote protein deposition in largemouth bass. This indirectly indicates an enhancement in the growth performance of largemouth bass, which may be correlated. Serum ALT, AST and AKP serve as crucial serum markers for identifying animal nutrition and health status (48, 49). The changes in the activities of enzymes, including ALT, AST, and AKP, are directly correlated with the extent of organ-specific cellular damage. As an extracellular enzyme, AKP plays a crucial role in various biological processes including growth, cell differentiation, protein synthesis and immune metabolism (50–52). Elevated serum AKP activity indicates compromised cell membrane permeability and integrity, resulting in cellular damage and reflecting the occurrence of host hepatobiliary inflammation (53, 54). The findings of this investigation demonstrate that dietary supplementation with CB can significantly modulate the serum AKP activity in largemouth bass, both increasing and decreasing it. Moreover, when liver inflammation occurs, CB has the potential to enhance phagocytic activity of immune cells against foreign bodies and thus protect the liver from toxic invasion. The results also indicates that dietary CB significantly enhances nutrient digestion, a finding corroborated in yellow catfish. ALT and AST are important transaminases in the human body, and their content can reflect the health status of the liver. Normally, the activity of serum aminotransferases is low. However, elevated levels of ALT and AST indicate liver damage (44). UN, or blood urea nitrogen, is not a protein; however, it serves as an indicator of the body’s protein metabolism status (54–57). Under the experimental conditions, it was observed that the control group exhibited elevated levels of serum AST, ALT, and UN, indicating potential hepatic inflammation or other pathological states. Notably, dietary supplementation of CB resulted in a significant reduction in serum AST, ALT and UN levels, indicating the hepatoprotective effect of CB on largemouth bass. However, further investigation is required to elucidate the underlying mechanism. Therefore, our objective was to identify immune and antioxidant genes in the liver of largemouth bass and comprehensively analyze the protective mechanism of CB against liver inflammation and disease states in this species.

The liver plays a crucial role in the response to oxidative stress and immune challenges (58). Oxygen free radical and lipid peroxidation reactions are integral components of the body’s metabolic processes (50, 52, 59, 60). Under normal circumstances, the two systems work in concert to maintain a multitude of physiological, biochemical, and immunological responses within the body (48, 51). SOD, POD, GSH-Px and CAT are crucial enzymes in the biological antioxidant defense system that play a vital role in scavenging reactive oxygen species within the body to safeguard cell membranes and nucleic acids (49, 61). T-AOC reflects the body’s compensatory ability to external stimuli and the status of free radical metabolism (62, 63). The MDA content serves as an indicator of the extent of cellular damage (63–66). The findings of this experiment indicate that dietary CB significantly enhances the hepatic activities of T-AOC, SOD, CAT, POD and GSH-Px in largemouth bass, which is consistent with the results obtained from a previous study on hybrid grouper (*♀Epinephelus fuscoguttatus × ♂ E. lanceolatu*) (60). It was also observed that dietary CB significantly reduced the MDA content in the liver of freshwater shrimp, which is consistent with the results of the study on yellow catfish (36), freshwater prawn (21), and Pacific white shrimp (67). The findings indicated that dietary supplementation of CB could significantly enhance the antioxidant enzyme activity in largemouth bass, decrease the level of MDA, suppress hepatic lipid peroxidation and safeguard against oxidative stress-induced damage. The incorporation of CB significantly augmented the antioxidant potential of largemouth bass, potentially ascribed to its pivotal role in scavenging highly reactive oxygen free radicals and producing antioxidant enzymes, thereby mitigating lipid peroxidation and effectively quenching free radicals, ultimately conferring cellular protection against oxidative damage (21).

Oxidative stress and inflammatory response are interrelated processes that significantly contribute to the body’s response to various stressors (68). The inflammatory response is initiated and modulated by pro-inflammatory cytokines (69, 70). Interleukin-8 (*IL-8*), a pro-inflammatory cytokine, stimulates the activation of macrophages and neutrophils, thereby promoting tissue regeneration in response to damage (71–73). Interleukin-1β (*IL-1β*) and tumor necrosis factor-α (*TNF-α*) primarily elicit inflammatory responses by modulating the expression of other cytokines (74). Interleukin-10 (*IL-10*) and transforming growth factor β (*TGF-β*) exert inhibitory effects on the proliferation, activation, and migration of T and B lymphocytes, thereby limiting the inflammatory response (75–78). Lysozyme (LZM) is a crucial antibacterial agent that can disrupt bacterial cell walls, stimulate the alternative complement pathway and phagocytic activity, and contribute to the body’s non-specific immune defense (74, 79–81). Toll-like receptors (TLRs) recognize invading microbial pathogens and initiate cellular signaling pathways. Toll-like receptor 22 (*TLR22*) and myeloid differentiation factor 88 (*MyD88*) are implicated in the regulation of inflammatory processes (82–84). Upregulation of *IL-6* and *TNF-α* expression may be attributed to tissue damage caused by infection or oxidative stress (85, 86). Under the current experimental conditions, dietary CB significantly suppressed the expression of hepatic *IL-8*, *IL-1β*, *TNF-α*, *MyD88* and *TLR22* genes in largemouth bass. Notably, the expression of *IL-10* and *TGF-β* genes was significantly upregulated in the liver of largemouth bass, while the activity of LZM was also significantly elevated. The findings indicated that dietary supplementation of CB could activate Toll receptors in largemouth bass, mediate *MyD88*-dependent signaling pathway, ultimately inhibit the release of proinflammatory cytokines *IL-8*, *IL-1β* and *TNF-α*, activate the acquired immune response, ultimately regulate immune function, enhance body resistance and mitigate liver inflammatory response. Similar studies have demonstrated that dietary CB significantly reduces serum levels of tumor necrosis factor-alpha (*TNF-α*) and increases interleukin-10 (*IL-10*) expression in children (87), markedly inhibits the expression of *IL-6* and *TNF-α* in chicken intestines (88), substantially elevates serum *IL-10* content and decreases the content of *IL-1β* in weanling piglets (89), and significantly reduces the expression of *IL-1β* and *IL-8* in yellow catfish (36). In summary, dietary CB can protect the liver from oxidative damage by regulating antioxidant enzyme activity and alleviate the hepatic inflammatory response by upregulating the expression of anti-inflammatory factors and inhibiting the expression of pro-inflammatory factors. Interestingly, the inclusion of CB in the diet resulted in a significant upregulation of *TLR22* expression in piglet ileum and promoted *MyD88* gene expression, which contrasts with the findings of this experiment. The expression of *MyD88* is modulated by its associated signaling cascade. In general, Toll-like receptors are the only ones stimulated by pathogens. *MyD88* is transcriptionally activated by anti-inflammatory factors through *NF-kB* family genes. Under the experimental conditions, when largemouth bass was exposed to oxidative stress, the liver underwent pathological changes and the body was stimulated by bacteria or toxins. Toll-like receptors (TLRs) mediated immune responses through the *MyD88*-dependent pathway. The inclusion of CB in the diet resulted in a significant increase in hepatic LZM activity and stimulated phagocytic function. The expression of pro-inflammatory cytokines *IL-8*, *IL-1β* and *TNF-α* was significantly reduced, while the genes for anti-inflammatory cytokines *IL-10 and TGF-β* were up-regulated, leading to suppression of the inflammatory response. The comprehensive analysis revealed that CB exerted significant effects on the expression of *TLR22* pathway-associated genes in largemouth bass. CB has the potential to modulate *TLR22* pathway-related factors, thereby mitigating inflammatory response and enhancing immune function.

Hypoxia is a common stressor experienced by aquatic animals in aquaculture, which can result in inhibited behavior and physiological metabolism of fish, ultimately leading to oxidative damage across various organs. Fish can enhance their antioxidant activities by increasing the activity of antioxidant enzymes. The dietary addition of CB significantly boosts the antioxidant capacity of tilapia, *Macrobrachium robertsoni*, *Penaeus vannamei*, and *Penaeus prefixus*. Reaffirming the findings of previous studies, the results of our current experiment demonstrate that dietary CB significantly enhances the activities of antioxidant enzymes in a similar manner. Interestingly, the results of the 3-hour hypoxic stress experiment on largemouth bass revealed that CB1, CB2, CB3 and CB4 had significantly lower CMRs than CB0 by 21.22%, 24.25%, 54.55% and 42.43% respectively, indicating a significant improvement in hypoxia stress survival rate due to dietary CB. The enhanced anti-hypoxic activity of CB in promoting survival may be attributed to the upregulated hepatic antioxidant enzyme activities (SOD, GSH-Px, CAT, POD) and decreased hepatic MDA concentration observed in this study, which ultimately improved the antioxidative status of largemouth bass by elevating hepatic T-AOC levels.

## 5 Conclusions

Dietary CB (1.5×10^9^ CFU/kg, CB3) can enhance the growth, antioxidant activity and hypoxic stress resistance of largemouth bass. By regulating GH/IGF-1 gene expression and inflammatory factors, as well as inhibiting TLR22/MyD88 signaling pathway, dietary CB can improve the growth performance and hypoxic stress resistance of largemouth bass.

## Data availability statement

The datasets presented in this study can be found in online repositories. The names of the repository/repositories and accession number(s) can be found in the article/supplementary material.

## Ethics statement

The animal study was reviewed and approved by Collaborative Innovation Center of Aquatic Sciences, Guangdong Key Laboratory of Animal Breeding and Nutrition, Institute of Animal Science, Guangdong Academy of Agricultural Sciences.

## Author contributions

PL wrote the paper; XC and BC performed the experiments; DH performed the experiments; KP performed the experiments; WH analyzed the data; JC designed the experiments; HZ conceived the experiments.
